# Patent Review of Lower Limb Rehabilitation Robotic Systems by Sensors and Actuation Systems Used

**DOI:** 10.3390/s23136237

**Published:** 2023-07-07

**Authors:** Cristina Floriana Pană, Dorin Popescu, Virginia Maria Rădulescu

**Affiliations:** 1 Department of Mechatronics and Robotics, University of Craiova, 200440 Craiova, Romania; cristina.pana@edu.ucv.ro; 2 Department of Automation and Electronics, University of Craiova, 200440 Craiova, Romania; virginia.radulescu@edu.ucv.ro

**Keywords:** sensor, actuator, patent, robot, rehabilitation, lower limb

## Abstract

Robotic systems for lower limb rehabilitation are essential for improving patients’ physical conditions in lower limb rehabilitation and assisting patients with various locomotor dysfunctions. These robotic systems mainly integrate sensors, actuation, and control systems and combine features from bionics, robotics, control, medicine, and other interdisciplinary fields. Several lower limb robotic systems have been proposed in the patent literature; some are commercially available. This review is an in-depth study of the patents related to robotic rehabilitation systems for lower limbs from the point of view of the sensors and actuation systems used. The patents awarded and published between 2013 and 2023 were investigated, and the temporal distribution of these patents is presented. Our results were obtained by examining the analyzed information from the three public patent databases. The patents were selected so that there were no duplicates after several filters were used in this review. For each patent database, the patents were analyzed according to the category of sensors and the number of sensors used. Additionally, for the main categories of sensors, an analysis was conducted depending on the type of sensors used. Afterwards, the actuation solutions for robotic rehabilitation systems for upper limbs described in the patents were analyzed, highlighting the main trends in their use. The results are presented with a schematic approach so that any user can easily find patents that use a specific type of sensor or a particular type of actuation system, and the sensors or actuation systems recommended to be used in some instances are highlighted.

## 1. Introduction

Humans, or hominins, possess three distinct features that set them apart from other primates: bipedal walking, evolved hands, and a larger brain capacity. While walking on two legs offers advantages, such as better mobility, it also has drawbacks, such as putting pressure on organs, causing spinal problems, and slower movement. Being unable to walk can also have significant psychological effects in modern society.

Unfortunately, various diseases and injuries can impact the lower limbs, including the ankle, knee, and hip. The lower limbs play a crucial role in an individual’s gait. One’s gait, rhythm, and posture can provide important information about their overall health. Medical professionals have identified multiple gait patterns that can indicate different ailments or health issues.

Maintaining a healthy posture and gait is essential for one’s overall well-being. A proper gait enables individuals to achieve functional independence, making detecting and analyzing gait disorders imperative to enhance rehabilitation efforts and prevent complications. Optimal recovery outcomes can be achieved by using rehabilitation, gait training, and assistive/recovery devices.

Robot-assisted lower limb rehabilitation and assistive technology have fascinated engineers and researchers, providing much-needed assistance to individuals suffering from illnesses or injuries. As a result, various robotic lower limb devices and exoskeletons have been proposed and developed, some of which are already available for commercial use.

Rehabilitation robotic systems are categorized based on the body part they aid in the recovery process. They are designed to assist with the upper limb, lower limb, hand, or entire body. The system’s purpose and number of degrees of freedom influence its design and manufacturing solutions. Additionally, there are two types of rehabilitation systems: active and passive, depending on the motor involvement of the patient during recovery activities.

Two options exist to place actuators: distally or directly at the joint level. The distal option requires a torque transmission mechanism to the joint, while the direct joint level option does not need it but may increase the inertia of the moving parts. Each position has its advantages and disadvantages to consider.

Rehabilitation robotic systems have utilized different actuation systems depending on the energy source, such as electric, pneumatic, or hydraulic. Each of these systems comes with its own set of benefits and drawbacks. Additionally, unconventional actuators, such as shape memory alloys, electroactive polymers, and magneto-rheologic fluids, have been tested and analyzed.

Various sensing technologies have been successfully incorporated into robotic rehabilitation systems. These sensors can measure limb movements, forces, torques, bio-signals, etc.

Assistive robotic systems, such as exoskeletons, are mechatronic systems that utilize mechanics, sensors, actuators, and control components. The specialized literature provides numerous reviews on the current state of rehabilitation systems for the lower limbs, specifically regarding their sensors and actuation systems. These reviews focus solely on experimental models developed by research teams or commercial models produced by companies.

As a novelty, our review is an in-depth study of the patents related to rehabilitation robotic systems for lower limbs from the point of view of the sensors and actuation systems used. The patents awarded and published between 2013 and 2023 were investigated, and the temporal distribution of these patents is presented. Our results were obtained by examining the analyzed information from the three public patent databases (Google Patents, Patent-Scope, Lens). The patents were selected so that there were no duplicates after several filters were used in this review. For each patent database, the patents were analyzed according to the category of sensors and the number of sensors used. Additionally, for the main categories of sensors, an analysis was conducted depending on the type of sensors used. Afterwards, the actuation solutions for rehabilitation robotic systems for lower limbs described in the patents were analyzed, highlighting the main trends in their use.

Through this review, the authors want to help researchers by recommending sensors and actuation systems to be used in robotic systems to recover the lower limb by easily and quickly finding patents that use a specific sensor or actuation system.

Compared to other reviews that have investigated book chapters, journals, and conference papers, our study analyzed patents, categorizing them according to different sensors or actuation systems used to show significant indications regarding the trends of research activity in this field.

The first selection of patents was based on two criteria: to be a rehabilitation robotic system according to the purpose of their use, namely if they were used for the recovery of the lower limb.

Investigating the technical solutions of sensors and actuation systems proposed for robotic systems for the recovery of the lower limb can be a helpful contribution to future projects. This review can also be a valuable source of information for researchers and therapists in their work.

It is known that there are differences between the patents and scientific papers published in journals or conference proceedings, for example, from the evaluation point of view or their purpose. Patent evaluations are carried out differently than journal publications, depending on the requirements imposed by the patent laws of a particular country.

There are numerous publications (reviews that investigate book chapters, journals, and conference papers) about technological advances and available solutions for robotic systems in the rehabilitation of the upper or lower limbs. In the following, we mention only a few of them and what analyses their authors have carried out. A review was presented by Ho Shing Lo et al. in 2012 [1], while Maciejasz et al. provided a comprehensive analysis of robotic systems for upper limb rehabilitation in 2014 [2], covering various areas such as aim, target group, type of assistance, mechanical design, control strategy, and clinical evaluation. Other notable review papers in this field include Blank et al.’s (2014) paper [3] on promoting patient engagement in therapy, Babaiasl et al.’s (2015) paper [4] on rehabilitation robots and their clinical outcomes, and Rehmat et al.’s (2018) paper [5] on the use of robotic exoskeleton systems, mechanical structures, and control strategies. Furthermore, Manna et al. conducted a comparative study of actuation systems in 2018 [6], while Sanjiuan et al. explored construction solutions based on cable transmission in 2020 [7]. Lastly, Sobiech et al.’s (2022) paper [8] focused on exoskeletons that can rehabilitate three joints: wrist and hand joints, elbow joints, and shoulder and clavicle joints.

Over time, there have been many scientific works whose authors presented the state of the art or reviewed what has been designed, developed, or tested as a robotic system for the recovery of the lower limb. In the order of their appearance, only a few are presented in this work. One of the first works that carried out a literature review on lower limb robotic rehabilitation, as well as on the challenges in this field, was the work of Inaki Dıaz et al. in 2011 [9]. A few years later, Young and Ferris, in 2017 [10], presented the state of the art and future directions for lower limb robotic exoskeletons from their perspective.

Eiammanussakul and Sangveraphunsiri, in 2018 [11], presented a lower limb rehabilitation robot in a sitting position with a review of training activities. Additionally, in 2018, Carpino et al. [12] presented a meta-analysis and state of the art assessing the effectiveness and costs of robot-mediated lower limbs rehabilitation. In 2019, Sanchez-Villamañan et al. [13] comprehensively reviewed mechanical design principles for compliant lower limb exoskeletons. Shi et al. [14] reviewed lower limb rehabilitation exoskeleton robots.

Hobbs and Artemiadis, in 2020 [15], conducted a review of robot-assisted lower limb stroke therapy. They highlighted unexplored paths and future directions in gait rehabilitation. In 2021, Chaichaowarat et al. [16] presented actuators and sensors for rehabilitation robotics, Zhou et al. [17] achieved a review on lower limb rehabilitation exoskeleton robots, and Rodríguez-Fernández et al. [18] presented a systematic review on wearable lower limb exoskeletons for gait training in neuromuscular impairments.

Numerous materials have been used for lower limb rehabilitation robotic systems, resulting in the design of various robotic exoskeletons for patient gait assistance. Hussain et al., in 2021 [19], highlighted the importance of the choice of materials in constructing exoskeleton robotic systems and the advantages and disadvantages of different materials and manufacturing methods. Their review showed that material selection strongly affects the weight and performance of the robotic exoskeleton.

Sarajchi et al., in 2021 [20], published a systematic review of the mechanical design, actuation type, control strategy, and clinical evaluation of wearable lower limb exoskeleton for children with cerebral palsy.

In 2022, Tiboni et al. [21] presented a review on sensors and actuation technologies in exoskeletons. Sierra et al. [22] presented kinematics, actuation, and sensing architectures for rehabilitation and assistive robotics. Neťuková et al. [23] presented the state of the art of lower limb exoskeleton sensors. Massardi et al. [24] published a review on the characterization and evaluation of human–exoskeleton interaction dynamics.

Mathew et al., in 2023 [25], presented a systematic review of technological advancements in signal sensing, actuation, control, and training methods for robotic exoskeletons for rehabilitation.

Usually, the key technical information is not described in the papers. Many of the solutions proposed in the papers are experimental models, which are not patented. There are quite a few commercial solutions on the market. For these reasons, this review of the existing patents for the sensor and actuation systems of robotic rehabilitation systems was carried out.

In our thorough research of the literature, we did not come across any detailed analyses of patents related to sensors and actuators in the past decade. While some reviews touch on this subject (sensors and actuators), they rely solely on published papers.

This paper is organized as follows: Section 2 describes the research method regarding the analysis of patents from the point of view of sensors and actuation systems used by rehabilitation robotic systems for the lower limb. Section 3 reports the results of the patent review of the three analyzed patent databases, while in Section 4, the authors discuss the results of the patent review of sensors and actuation systems used by rehabilitation robotic systems for the lower limbs. Section 5 summarizes the most significant conclusions of this review paper.

## 2. Materials and Methods

### 2.1. Data Collection

Depending on the use of these devices for recovering the lower limbs, we can catalog them in robotic assistance and therapeutic exoskeletons for rehabilitation. The first category of robotic lower limb exoskeletons allows users to complete movements that they could not perform independently with the additional help of crutches. On the other hand, the second category is intended for the realization of therapeutic exercises by the user by training the muscles and the nervous system. However, some devices are combinations of the two categories, which is helpful for treating patients with locomotor difficulties by increasing their current physical capacities.

The data used by us in this study were collected from three online patent databases: Google Patents, Patent-Scope, and Lens, from 1 January 2013, to 31 March 2023, using the following search terms: (Exoskeleton) AND (Robot) AND (Walk OR Gait) AND ((Leg OR Lower) AND (Limb OR Extremity)) AND (Rehabilitation).

### 2.2. Analysis Method

#### 2.2.1. Inclusion Criteria

All patterns must be:

Filter 1: Published in or translated to English, and directly related to lower limb rehabilitation robotic systems.

Filter 2: Related to the International Patent Classification (IPC) A61H3/00 (appliances for aiding patients or disabled persons to walk using orthopaedical devices for correcting deformities of, or supporting, limbs (A61F 5/0102), knees (A61F 5/0106), or feet or ankles (A61F 5/0111).

Filter 3: Describes the design of a robotic system (exoskeleton) and contains data on the sensors and/or actuation system used.

Filter 4: Registered in any patent office in any country.

Filter 5: Awarded and published.

#### 2.2.2. Exclusion Criteria

All patterns must not be:

Filter 6: Related to rehabilitating the upper limbs (hand or arm).

Filter 7: Inactive, suspended, or pending legal status.

Filter 8: The publication date of the patent was not before 1 January 2013.

Filter 9: Methods that do not contain the description of the exoskeleton, sensors, and/or the actuation system.

Filter 10: Appear in duplicate, triplicate, etc.

#### 2.2.3. Search Methodology

We used a long-term analysis technique to determine the types of sensors and actuation systems used in the patents published in the last decade. Then, by comparison, we determined which are predominant.

With the above keywords and Filters 1, 2, 4, 6, and 7, we initially retrieved 5012 patents on Google Patents, 39 patents on Patent-Scope, and 1029 patents on Lens. Then, after applying Filters 3, 5, and 8, we obtained 1615 patents from Google Patents, 27 patents from Patent-Scope, and 122 patents from Lens. Finally, after using Filters 9 and 10, we obtained 283 patents on Google Patents; the exact number remained on Patent-Scope, and 67 patents were published on Lens.

## 3. Results

Based on our thorough analysis of the three platforms, we have compiled conclusive results categorized into two sections: sensors and actuation systems.

### 3.1. Sensors

In general, the senses of a robotic system are realized with the help of sensors that allow for the evaluation of the environment in which it operates. Based on the data collected from the sensors, the robot can adjust its actions. Over time, both researchers and engineers have contributed to the development of many types of sensors. Depending on the needs, a limited number of sensors or a plurality of sensors can be used.

We have compiled a comprehensive list of commonly used sensors, arranged alphabetically for easy reference. The types of sensors included in this list are:

- Acceleration: Used to determine the acceleration of the leg/foot.
- Bio-sensor: A highly sophisticated device that effectively gathers data on a user’s biological information. It is designed to measure physical or chemical reactions with utmost precision by generating signals corresponding to an analyte’s concentration in the response. This sensor can be:
  ➢ Electromyogram (EMG): Used to measure muscle tension;
  ➢ Electrodermal activity (EDA): Used to measure changes in perspiration rate;
  ➢ Finger pulse: Used to measure blood pressure and heartbeat;
  ➢ Electroencephalogram (EEG): Used to measure electrical brain activity;
  ➢ Electrocardiogram (ECG/EKG): Records electrical signals passing through the heart;
  ➢ Electrooculography (EOG): Used to graphically record the potential difference between the cornea and the posterior pole during dark adaptation when the eyes move from left to right at a standard distance;
  ➢ Body temperature;
  ➢ Heart rate monitors (HR), etc.
- Distance/detection: Used either to determine the location in the space of the exoskeleton or for detection. Various sensing technologies are available, including ultrasonic, infrared (IR), LIDAR, time-of-flight (ToF), GPS, radar, and proximity sensors;
- Encoder: Typically used in motion control to provide information about the position, speed, and direction. An array of sensor types exists, including linear, rotary, angle, absolute, incremental, optical, magnetic, capacitive, and quadrature sensors;
- Force: Used to measure the amount of force applied. A diverse range of force sensors catering to a variety of applications is available, including load cells (pneumatic, hydraulic, piezoelectric crystal, inductive, capacitive, magnetostrictive, strain gage), force sensing resistors (FSRs), optical force sensors, ultrasonic force sensors, force-torque (F/T), tension–pressure, tension, pulling, and frictional sensors;
- Force–torque (F/T): Used to measure the amount of reaction force produced by an object generating torque;
- Hall effect: Measures the voltage fluctuations when the device is exposed to a magnetic field;
- Inertial measurement unit (IMU): Gauges the exoskeleton’s orientation and tracks movements across various axes. Different types of sensors are available, including gyroscopes, accelerometers, and magnetometers, which can be utilized for various purposes;
- Position or motion sensor: Adept at detecting and transforming an object’s movement into signals that can be effectively harnessed for processing, transmission, or control motion objectives. Numerous sensor types are available, such as active detectors, passive infrared sensors, microwave sensors, ultrasonic sensors, tomographic motion detectors, video cameras, gesture detectors, and dual-technology motion sensors;
- Potentiometer: Used to provide feedback on a specific voltage value, which is then used to detect the motor’s position;
- Pressure: Commonly utilized for measuring air or fluid pressure and foot pressure on the sole/in-shoe or platform;
- Tension: Used to measure the tension force of the pneumatic muscle.
- Tilt: Used to detect orientation or inclination;
- Torque: Measures the torque electric motors produce on the joints along the sagittal plane;
- Other: This group comprises infrequently employed sensors (only once) in the patents reviewed, including audio alarm, flex, temperature, current, and voltage sensors.

#### 3.1.1. Google Patents Platform

**Figure 1:**
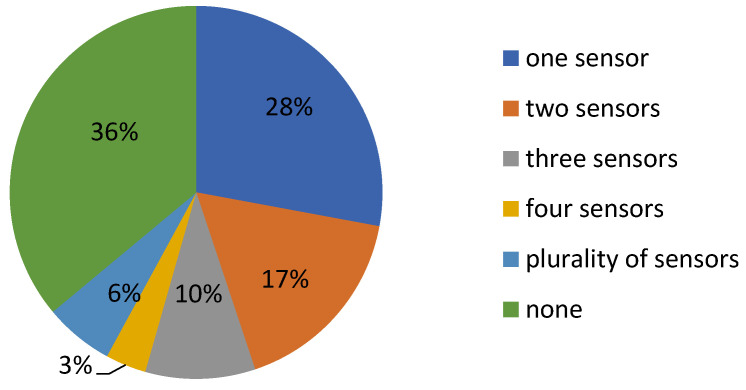
Distribution of patents on the Google Patents platform, according to the number of sensors used.

The distribution of patents based on the number of sensors they utilize is illustrated in Figure 1.

In analyzing Figure 1, it is evident that most patents (36%) did not specify the use of the sensors or only mentioned them in a generic context. These patents primarily emphasize the construction or actuation aspects of the devices. A substantial portion of patents (28% of the total) utilized only one type of sensor. Conversely, the patents which utilized four types of sensors had the lowest percentage, at 3%. Patents that used more than five sensors were established for a plurality of sensors, making up a percentage of 6%.

**Figure 2:**
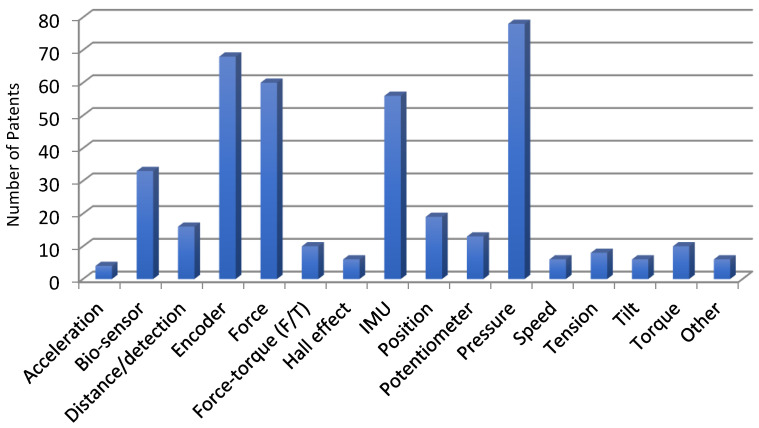
The distribution of patents on the Google Patents platform depends on the types of sensors used.

Table 1 presents the bibliographic references according to the type of sensor used. Bibliographic references where sensors were not used or given, such as patents [26,27,28,29], were removed. In Figure 2, the distribution of the patents is displayed according to the type of sensor used.

Pressure actuation-based sensors were the most commonly used, with nearly 30% of patents including them in their components. According to Table 2, foot plantar sensors were the most frequently used pressure sensors. This was expected, as a significant number of exoskeletons follow the step cycle of users.

When transmitting and transforming information, the encoder plays a crucial role. It is interesting to note that nearly 24.03% of the exoskeletons contained this element. For more detailed information on the usage of encoders, please refer to Table 3.

Proper handling of the force sensor is essential. Table 4 shows the different types of forces that are employed. Among the 283 projects found on Google Patents, 21.20% involved the utilization of force sensors.

**Figure 3:**
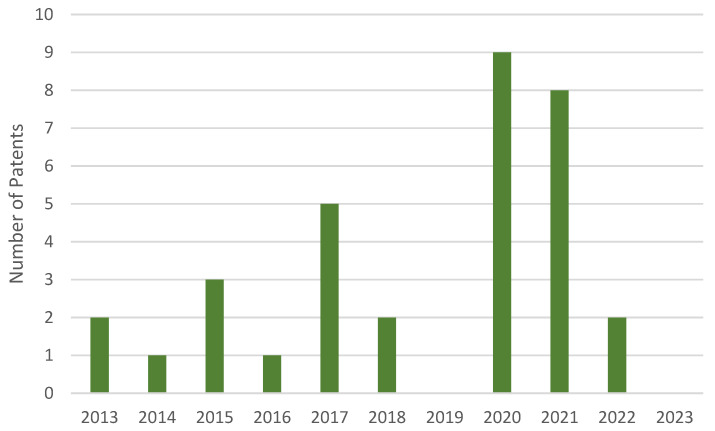
Time distribution of patents that use bio-sensors on the Google Patents platform.

Based on our analysis of Figure 3, it is evident that bio-sensors have been increasingly utilized, as evidenced by their presence in about 23 patents. These sensors offer specific advantages in certain applications, making them an attractive option for many exoskeletons.

#### 3.1.2. Patent-Scope Platform

**Figure 4:**
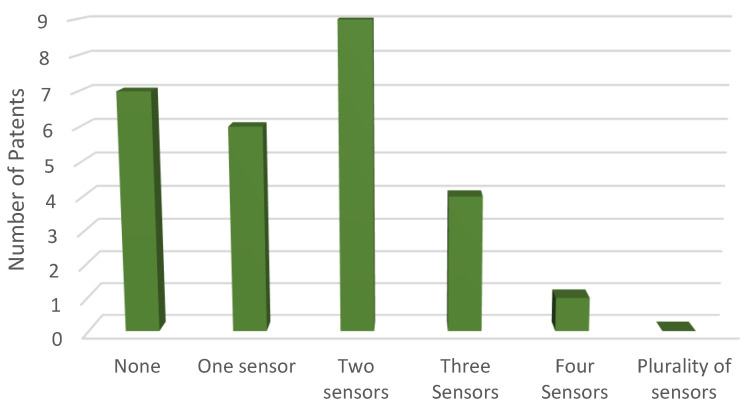
Distribution of patents on the Patent-Scope platform, according to the number of sensors used.

Figure 4 and Table 5 illustrate the patent distribution based on the number of sensors utilized.

In analyzing the Patent-Scope platform’s database, it was uncovered that around 70.36% of the exoskeletons possess at least one sensor, with a maximum of three sensors. Moreover, exoskeletons with more than four sensors, which are considered complex, account for an extremely low percentage of below 3.70%.

Table 6 presents the bibliographic references according to the type of sensor used. As in the case of the Google Patents platform, bibliographic references where sensors were not used or presented were removed.

**Figure 5:**
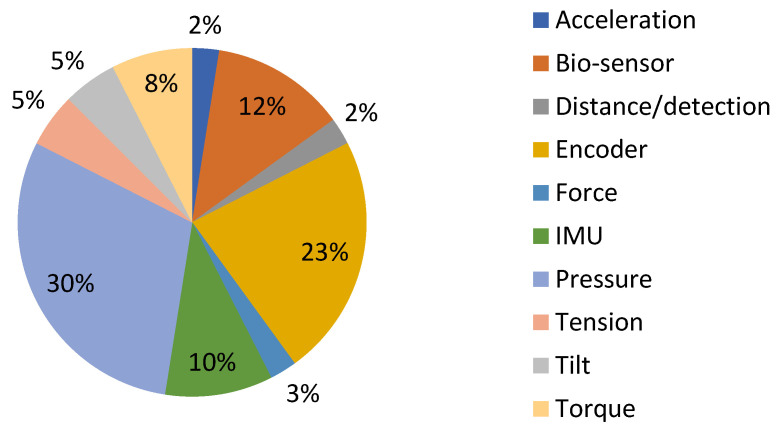
Distribution of patents on the Patent-Scope patents platform, according to the type of sensors used.

Based on the data analysis carried out on Patent-Scope, pressure sensors are the most widely utilized type of sensors. As depicted in Figure 5, approximately 30% of patients have employed at least one pressure sensor, making it the most common category. The second most frequently encountered category was exoskeletons involving encoders, followed by bio-sensors.

The results of the pressure sensors and encoders on the Patent-Scope platform are presented in Table 7 and Table 8, respectively.

After analyzing the Patent-Scope database, it was clear that only 1 out of 27 utilized force sensors, while 5 patents impressively incorporated bio-sensors into their design.

#### 3.1.3. Lens Platform

**Figure 6:**
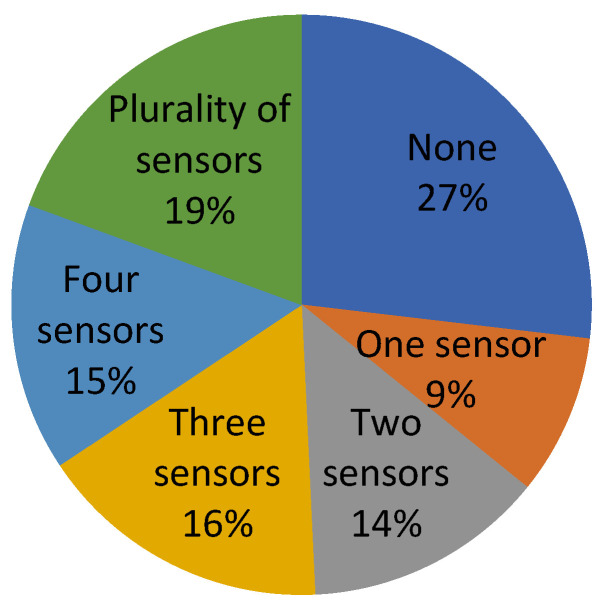
Patents on the Lens platform, distributed according to the number of sensors used.

The distribution of patents based on the number of sensors they utilize is illustrated in Figure 6.

**Figure 7:**
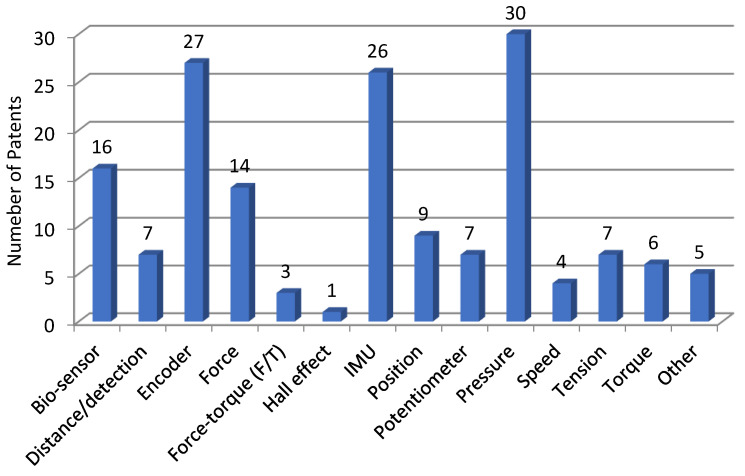
Distribution of patents on the Lens Patents platform, depending on the type of sensors used.

Table 9 presents the bibliographic references according to the type of sensor used. As in the case of the above platforms, bibliographic references where sensors were not used or given were removed. Figure 7 shows the distribution of patents according to the sensor type used.

The results of the pressure sensors, encoders, and force on the Lens platform are presented in Table 10, Table 11 and Table 12, respectively.

### 3.2. Actuating System

The actuation system is a crucial component of achieving movement in exoskeletons. The proper selection of actuators depends on the anatomical joints of the human lower limb. Since certain joints, such as the ankle, have multiple degrees of freedom, independent actuators can only provide one. As a result, the type of actuation required is the deciding factor in selecting the appropriate actuator.

Evaluating the precision of a joint movement technique is crucial when selecting the appropriate method. Various methods, such as motor or servo-driven joints, pneumatic or hydraulic systems, and air muscles, can be used to move joints. Additionally, combining active and passive techniques, such as spring-based systems, may be advantageous. Finally, factors such as the required force, speed range, and available space influence the choice of technique. Rest assured that the optimal technique can be determined carefully considering these factors.

We have categorized the actuating systems into two groups: conventional (including mechanical, electric, hydraulic, and pneumatic) and unconventional (such as artificial muscle, serial elastic, and others). Furthermore, we have meticulously arranged a comprehensive and easy-to-use list of frequently utilized actuating systems for quick reference.

#### 3.2.1. Google Patents Platform

**Figure 8:**
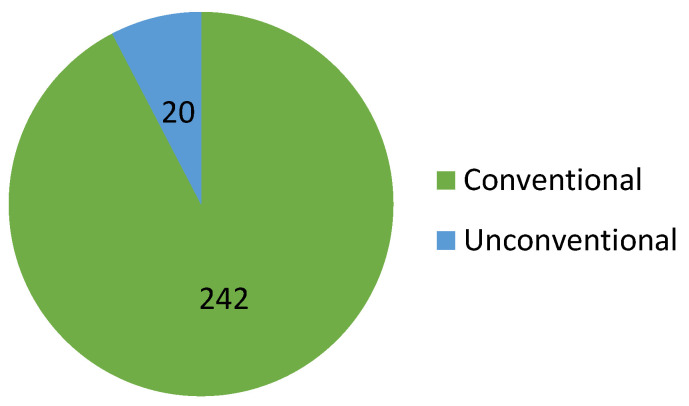
The distribution of patents on the Google Patents platform, according to the types of actuators used.

Regarding the Google Patents platform, Figure 8 provides a detailed breakdown of the percentage of actuation systems used in the patents, including conventional and unconventional actuators.

**Figure 9:**
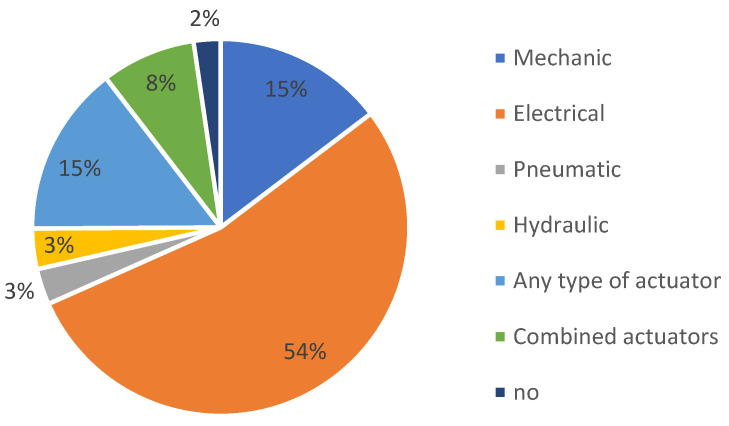
The distribution of patents on the Google Patents platform, according to the types of conventional actuators used.

It is important to note that when we say “any type of actuator”, we specifically refer to exoskeletons adaptable to various conventional actuation methods (Figure 9). Similarly, “combined actuators” means any possible combination of conventional actuators.

**Figure 10:**
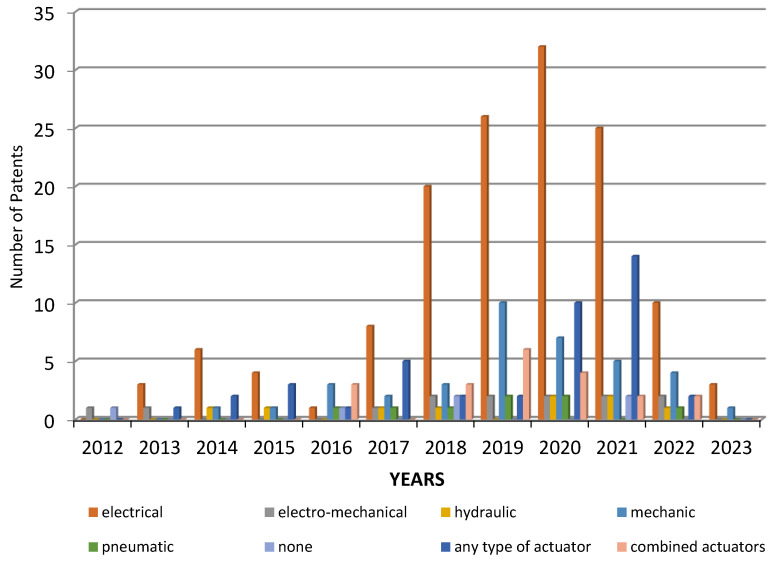
Distribution of patents over time on the Google Patents platform, depending on the types of actuators used.

Figure 10 (Google Patents platform) shows the year-by-year representation of actuator types.

Table 13 presents the bibliographic references according to the type of actuating system used. Bibliographic references where actuating systems were not used or given were removed.

**Figure 11:**
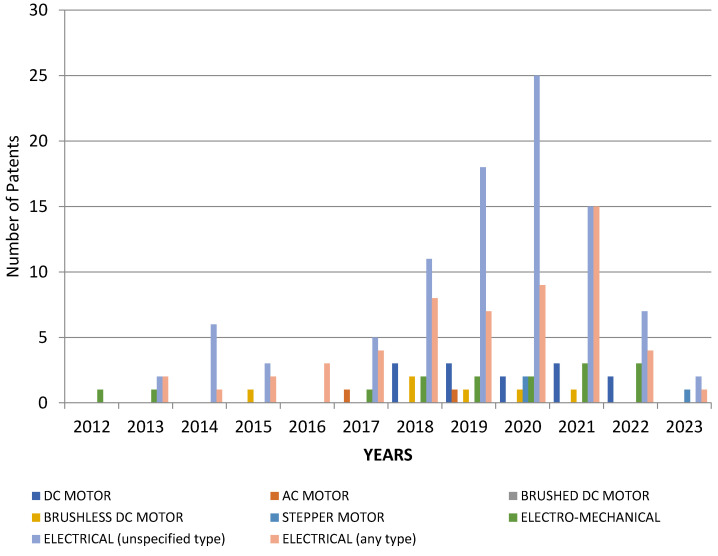
Distribution of patents over time on the Google Patents platform, depending on the types of electric actuators used.

The distribution of electric-type actuators is clearly shown in Figure 11.

#### 3.2.2. Patent-Scope Platform

**Figure 12:**
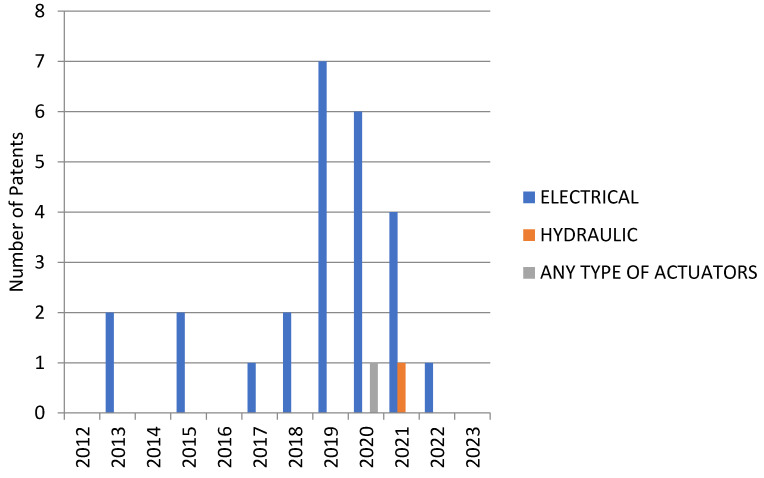
The distribution of patents in time on the Patent-Scope platform, depending on the types of actuators used.

Figure 12, regarding the Patent-Scope platform, provides a comprehensive evaluation of the actuator types used year by year.

Table 14 presents the bibliographic references according to the type of actuating system used.

**Figure 13:**
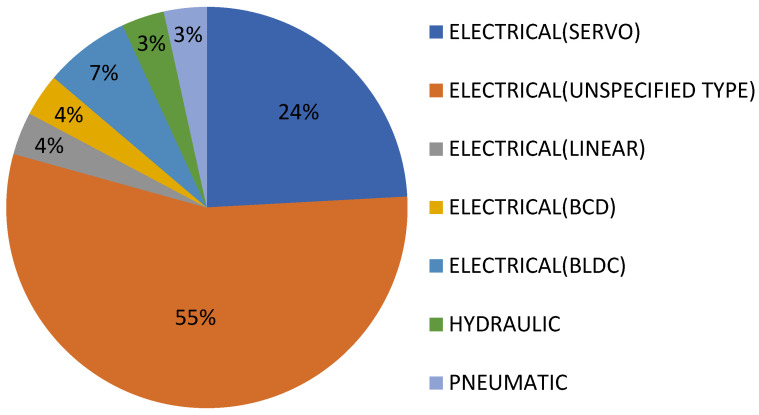
Distribution of patents on the Patent-Scope platform, depending on the type of electric actuators used.

The distribution of electric-type actuators is clearly shown in Figure 13.

#### 3.2.3. Lens Platform

**Figure 14:**
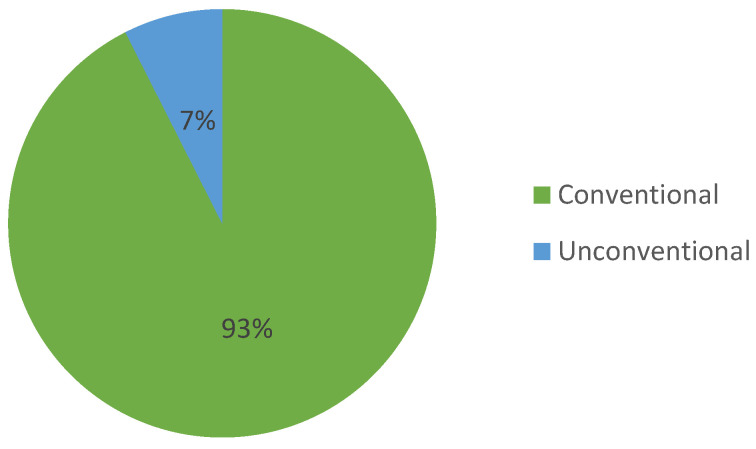
The distribution of patents on the Lens platform, according to the types of actuators used.

Regarding the Lens platform, Figure 14 provides a detailed breakdown of the actuating systems used in the patents, including conventional and unconventional actuators.

**Figure 15:**
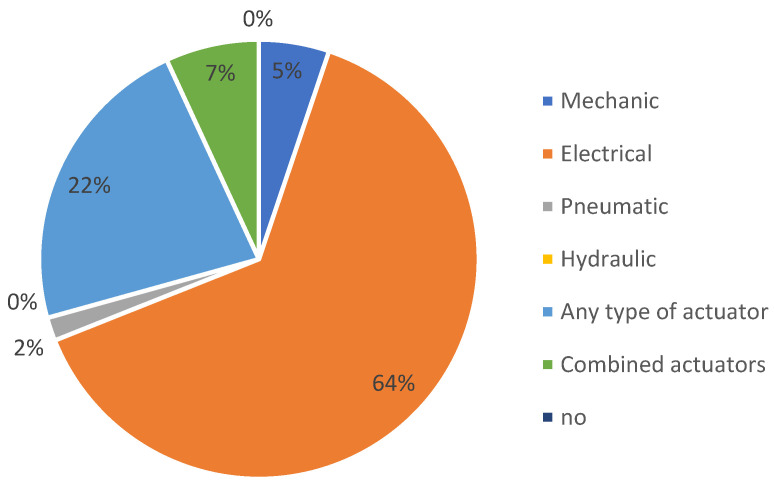
The distribution of patents on the Lens platform, according to the types of conventional actuators used.

Figure 15 provides a detailed breakdown of the percentage of the actuation systems used in the patents on the Lens platform.

Table 15 presents the bibliographic references according to the type of actuating system used.

**Figure 16:**
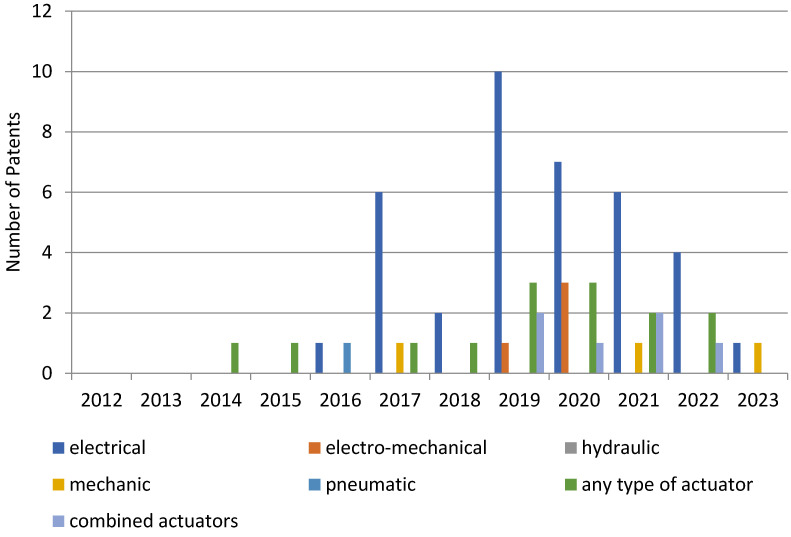
The distribution of patents over time on the Lens platform, depending on the type of actuators used.

Figure 16 offers a comprehensive yearly evaluation of various actuator types on the Lens platform.

## 4. Discussion

Recovering the ability to walk after lower limb injuries requires considerable time and physical effort from specialized medical personnel, therapists, physiotherapists, and patients. In light of this, implementing robotic rehabilitation for the lower limbs is a significant advantage towards achieving this goal [402]. Robotic exoskeleton systems have proven extremely beneficial for patients with spinal cord injuries [403,404], stroke [20,405], cerebral palsy [406], traumatic brain injuries [407], Parkinson’s disease [408], multiple sclerosis [409], elderly care [410], postoperative care [411], and orthopedics [412].

The specialized literature presents a wide range of research and evaluations on lower limb exoskeletons specially designed for rehabilitation. Numerous studies have thoroughly scrutinized the exhibition of specific exoskeletons [413,414,415,416,417] or comprehensively analyzed published papers across diverse platforms [12,21,24,418,419,420,421,422].

Based on our study findings, we can draw dependable conclusions regarding the analysis results obtained from the three platforms.

**Figure 17:**
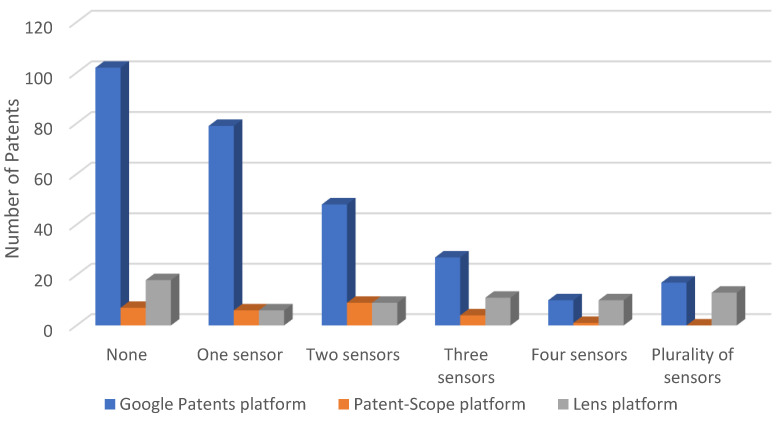
Distribution of patents on the three platforms, depending on the number of sensors used.

In Figure 17, the data exhibit the number of sensors employed in the exoskeletons assessed on the three platforms. While certain patents did not mention or integrate sensors, it is apparent that the utilization of sensors was tailored to fit the particular requirements of the rehabilitation process. Notably, at least one sensor was deemed essential in overseeing the rehabilitation procedure, signifying that inventors integrated sensors only where necessary. Consequently, it can be deduced that using sensors was prioritized for optimal efficiency.

**Figure 18:**
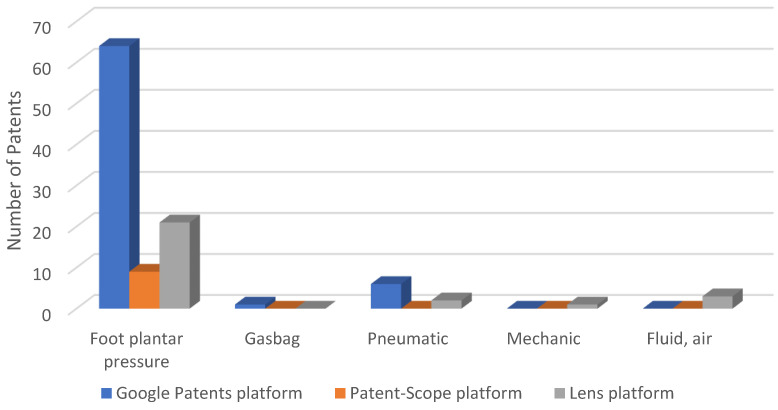
Distribution of the patents, according to the number of pressure sensors used.

The use of sensors in developing exoskeletons is a topic of great interest. Among the various types of sensors, foot plantar pressure sensors have gained much attention and have been extensively used in many patents analyzed across all three databases. This is especially true for patents on designing exoskeletons to aid gait rehabilitation. More detailed information on the distribution of patents, according to the pressure sensors used, can be seen in Figure 18.

After analyzing data from three different platforms, we can confidently assert that the electric actuation system reigns supreme in creating exoskeletons. Our findings align with those published in the specialized literature on this subject. Electric motors have made considerable progress in recent years, with various sizes and performances now widely available. They are more cost-effective than other actuators and remarkably compact. Furthermore, controlling them has become a much simpler process. Moreover, according to the statistical analyses described in [21,24,422], it is indisputable that electric motors are the most used drive mechanism in the literature.

Our study confirms what was expected, that is, most of the lower limb rehabilitation robotic systems proposed by the authors of the analyzed patents are electrically operated due to easy energy storage and supply. The advantages of electric actuators are a great variety of types and sizes, a good control accuracy, a good reliability. The most used electric actuators are brushed and brushless DC motors.

**Figure 19:**
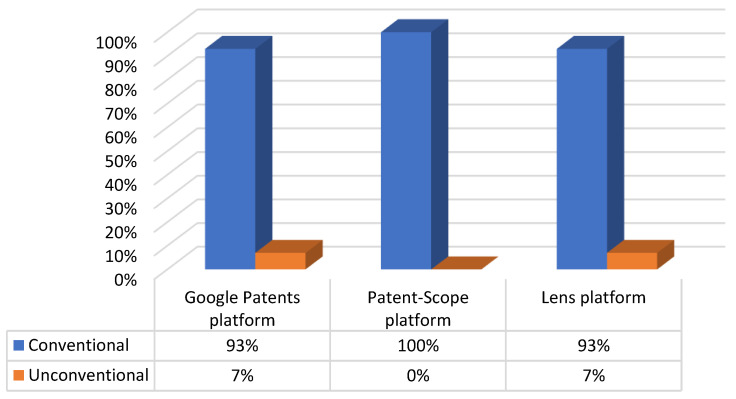
Percentage distribution of patents, according to the type of actuation system used.

An Important finding that cannot be ignored is that unconventional actuation techniques are not frequently utilized in patents (Figure 19). While unconventional actuation may be an option for some applications, it has its limitations, such as generating much smaller forces, which leads to instability in terms of the view of the kinematic/dynamic behavior that maintains the motion. Conventional actuation remains the preferred option, despite the low power-to-weight ratio and increasing gear sizes. This is due to its ability to generate higher forces and provide greater stability.

The authors of the patents have proposed different actuation systems for lower limb rehabilitation robotic systems. They have proposed electric, pneumatic, or hydraulic sources as a source of energy. Each of these systems comes with its own set of advantages and disadvantages. For the actuation of lower limb rehabilitation robotic systems, compared to the authors of the conference/journal papers, the authors of the analyzed patents have proposed unconventional actuators, such as shape memory alloys, electroactive polymers, and magneto-rheological fluids. Indeed, the number of these patents proposing unconventional actuators is very small.

According to a review conducted by Pamungkas et al. in [420], recent studies on prototype lower limb exoskeletons have revealed that certain exoskeletons feature actuated joints, while others utilize a combination of actuators that target multiple joints. While lower limb exoskeletons that mimic human biomechanics enable users to move more naturally, they tend to be more intricate and unwieldy. To reduce weight, some exoskeletons incorporate passive actuators instead of active ones, which comes at the cost of decreased controllability.

An intriguing observation can be made based on the results acquired from all three platforms. The years 2019 and 2020 saw the highest number of patent publications, with electric actuating systems predominating.

The actuation system has a fundamental role in the effectiveness of the rehabilitation robotic system because it influences the main functional characteristics of the system, for example, stability, control precision for the position, generated forces/torques, and safety. In addition, the chosen actuation system significantly influences the rehabilitation robotic system’s dimensions, weight, and rigidity.

**Figure 20:**
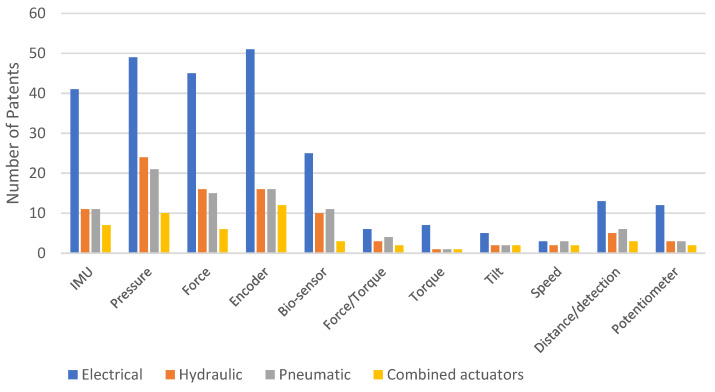
Distribution of patents on the Google Patents platform, according to the types of sensors used and depending on the actuating system.

**Figure 21:**
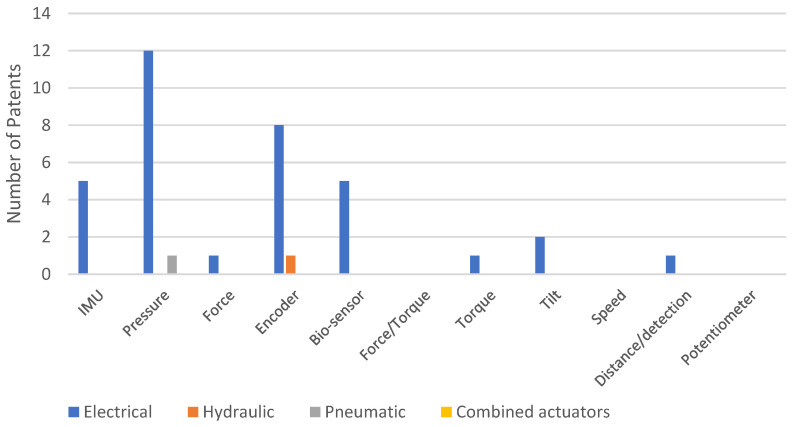
Distribution of patents on the Patent-Scope platform, according to the types of sensors used and depending on the actuating system.

**Figure 22:**
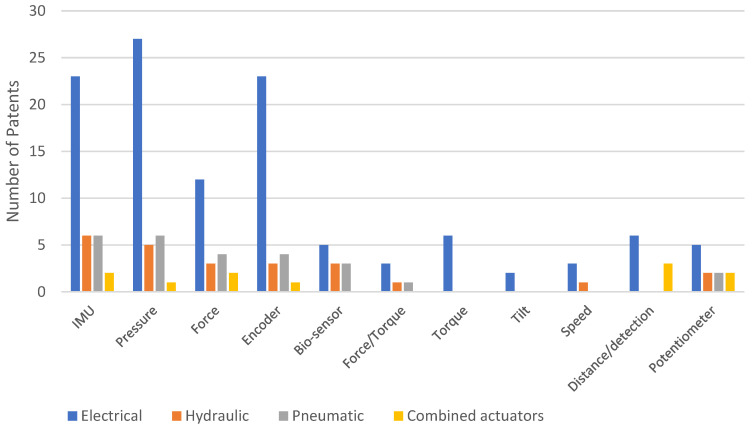
Distribution of patents on the Lens platform, according to the types of sensors used and depending on the actuating system.

**Figure 23:**
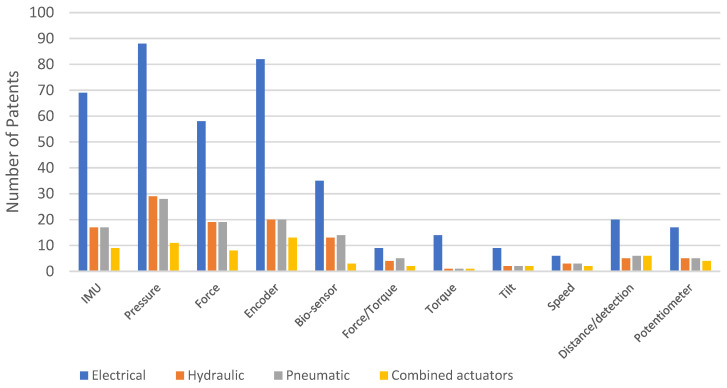
Distribution of patents on all three platforms, according to the types of sensors used and depending on the actuating system.

We also analyzed the distribution of patents for different types of sensors based on the actuation system used in exoskeletons across each platform. Figure 20 shows the distribution of patents on Google Patents, Figure 21 on Patent-Scope, and Figure 22 on Lens. Figure 23 illustrates the overall distribution of patents based on the sensor and actuation type.

Based on Figure 23, the sensors most frequently used for the four types of actuators, in hierarchical order, are pressure, encoders, IMU, force, and bio-sensors. However, when examining the platforms separately (as shown in Figure 20, Figure 21 and Figure 22), there are variations in this hierarchy. For instance, the order on the Google Patents platform is encoders, pressure, force, IMU, and bio-sensors, while the order is different on the other two platforms. On the Patent-Scope platform, the order is pressure, encoders, bio-sensors, IMU, and force, and on the Lens platform, the order is pressure, encoders and IMU (with the same number), force, and bio-sensors.

Cross-analyses were performed to highlight possible correlations between the actuation systems and the sensors used by the patent’s authors. Identifying these correlations can provide helpful information on the technology choices made for different types and uses of rehabilitation robotic systems for the lower limb and the outcomes achieved.

**Figure 24:**
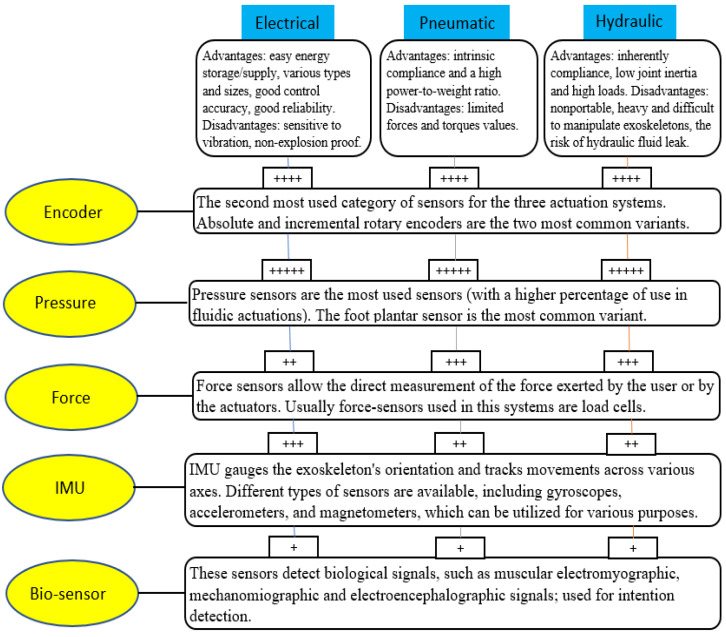
Features of sensors and actuation systems and recommendations of use.

This review aimed to help researchers by making recommendations regarding sensors and actuation systems to be used in robotic systems to recover the lower limb by easily and quickly finding patents that use a specific sensor or actuation system. In Figure 24, we highlight the main advantages and disadvantages of the actuation systems (electrical, pneumatic, and hydraulic) and some characteristics of the main categories of sensors used according to their use percentage. We considered only the first five sensor categories (encoder, pressure, force, IMU, and bio-sensor). The other sensors follow with quite lower percentages. We also tried to make recommendations for using sensors (+++++, the most used; +, the least used) depending on the type of actuation system used.

In multi-sensor systems, encoders, pressure sensors, force/torque sensors, and IMU and EMG sensors are usually used. By sensor fusion, a whole information acquisition system is created.

The sensors used for capturing the biomechanics of lower limb movements can be wearable or nonwearable by human subjects. Each type of sensor has its advantages and disadvantages. It is also important if the subject uses or does not use a rehabilitation robotic system.

An image processing-based system is one of the most used nonwearable sensor-based systems for motion capture, which is most suitable for gait analysis. For example, we used a VICON system (with 14 Vicon T10 video cameras) in a laboratory environment, which allowed for a video analysis of the subject’s movement, determining the parameters of interest of the movement of the lower limb. We obtained excellent results for each joint movement’s position, speed, and acceleration when the subject did not use a rehabilitation robotic system. When the subject/patient used a rehabilitation robotic system, it was more difficult to use an image processing-based system because the elements of the rehabilitation robotic system could cover some joints.

An advantage of wearable sensors is that they can be used indoors (in the laboratory) and in outdoor environments. IMU is the most commonly used wearable sensor (by human subjects) to measure human kinematics/kinetics. If we analyze the capturing of the biomechanics of lower limb movements when using the robotic rehabilitation systems with sensors of this system, then the most used sensors are encoders. EMG sensors are the most used noninvasive wearable sensors to evaluate muscle functions.

### Limitations of this Study

Despite some limitations due to a lack of specific details on sensors and actuators in certain cases, this study thoroughly examined lower limb exoskeleton patents and produced results that align with the existing specialized literature and devices currently available. The collected data provide a comprehensive understanding of the subject matter.

## 5. Conclusions

This study thoroughly evaluated the sensors and actuators used in rehabilitation robotic technology for lower limb recovery by analyzing only patents published between January 2013 and March 2023. Compared to other reviews that have investigated book chapters, journals, and conference papers, our study examines patents, categorizing them according to different sensors or actuation systems used to show significant indications regarding the trends of research activity in this field.

Our results were obtained by examining the information from the three public patent databases (Google Patents, Patent-Scope, and Lens). The review was performed on 283 patents from Google Patents, 27 from Patent-Scope, and 67 from Lens. The patents were selected so that there were no duplicates after several filters.

The aim was to identify areas for improvement and highlight the benefits of integrating this advanced technology. Through this review, the authors want to help researchers by recommending sensors and actuation systems to be used in robotic systems to recover the lower limb by easily and quickly finding patents that use a specific sensor or actuation system.

A cross-analysis was conducted to find possible connections between the actuators and sensors utilized by the patent authors. This can help in determining the suitable technology options for various types and applications of lower limb rehabilitation robotic systems, as well as the results achieved.

The authors believe their research can provide valuable insights for scientists and researchers to develop next-generation systems that effectively address mobility challenges. The final goal of the work was to create an image of the main solutions of actuation systems and sensors used by the authors of the patents for rehabilitation robotic systems of the lower limb, as completely as possible.

The patent activity related to rehabilitation robotic systems for the lower limb, awarded from 2013 to 2023, was significant and addressed various topics and issues. Given the complexity of these systems and due to patient safety requirements, there are many design challenges still unsolved or for improvement. Some examples are the dimensions and portability of the rehabilitation robotic system, the control of this system to increase the efficiency of recovery, and the safety and comfort of the patient. In this context, the authors plan to investigate the control methods employed in patented rehabilitation robotic systems.

## Figures and Tables

**Table 1:** Bibliographic references of sensors on the Google Patents platform, in alphabetical order.

Sensor	References
Acceleration	[30,31,32,33]
Bio-sensor	[34,35,36,37,38,39,40,41,42,43,44,45,46,47,48,49,50,51,52,53,54,55,56,57,58,59,60,61,62,63,64,65,66]
Distance/detection	[34,37,48,51,59,67,68,69,70,71,72,73,74,75,76,77]
Encoder	[30,35,36,37,41,44,45,48,51,56,59,60,62,63,70,78,79,80,81,82,83,84,85,86,87,88,89,90,91,92,93,94,95,96,97,98,99,100,101,102,103,104,105,106,107,108,109,110,111,112,113,114,115,116,117,118,119,120,121,122,123,124,125,126,127,128,129,130]
Force	[30,34,36,37,39,41,43,45,47,51,52,56,57,58,63,66,67,68,69,77,80,82,84,85,86,92,95,96,97,106,112,115,116,131,132,133,134,135,136,137,138,139,140,141,142,143,144,145,146,147,148,149,150,151,152,153,154,155,156,157]
Force–torque (F/T)	[46,48,62,90,91,93,158,159,160,161]
Hall effect	[60,67,125,126,162,163]
IMU	[30,34,35,36,40,41,42,44,45,46,48,51,53,55,56,59,61,62,63,70,71,73,74,75,80,81,82,84,88,89,92,97,98,99,100,104,107,125,126,127,131,137,158,164,165,166,167,168,169,170,171,172,173,174,175,176]
Position	[32,34,36,39,45,48,51,59,66,72,75,77,91,113,139,140,173,177,178]
Potentiometer	[41,44,45,46,56,62,63,68,77,82,86,89,98]
Pressure	[31,34,36,38,39,44,46,53,56,66,67,70,72,75,77,78,79,80,81,84,89,90,91,96,100,101,103,104,107,109,111,112,113,115,116,119,120,121,130,131,139,140,142,144,150,152,155,160,165,167,168,169,171,177,178,179,180,181,182,183,184,185,186,187,188,189,190,191,192,193,194,195,196,197,198,199,200,201]
Speed	[32,36,78,85,131,139]
Tension	[31,41,84,99,160,183,202,203]
Tilt	[36,78,84,95,124,204]
Torque	[37,77,83,100,115,129,134,153,156,205]
Other	[33,41,82,158,206,207]

**Table 2:** The distribution of the use of pressure sensors in the patents on the Google Patents platform, where P represents the total number of patents, and N is the number of patents that use the respective sensor.

Type of Sensor	P = 283
Pressure sensor, N (P%)	78 (27.56)
Foot plantar pressure, N (P%)	71 (25.09)
Gasbag, N (P%)	1 (0.35)
Pneumatic, N (P%)	6 (2.12)

**Table 3:** The distribution of the use of encoders in the patents pm the Google Patents platform, where P represents the total number of patents, and N is the number of patents that use the respective sensor.

Type of Sensor	P = 283
Encoder, N (P%)	68 (24.03)
Encoder (angle), N (P%)	51 (18.02)
Encoder (unspecified type), N (P%)	14 (4.95)
Encoder (linear, rotary), N (P%)	2 (0.70)
Encoder (magnetic, photoelectric), N (P%)	1 (0.35)

**Table 4:** The distribution of the use of force sensors in the patents on the Google Patents platform, where P represents the total number of patents, and N is the number of patents that use the respective sensor.

Characteristic	P = 283
Force sensors, N (P%)	60 (21.20)
Force (unspecified type), N (P%)	17 (6.00)
Force (FSR), N (P%)	16 (5.65)
Force (load cell), N (P%)	23 (8.13)
Force (pulling), N (P%)	4 (1.41)

**Table 5:** The percentage distribution of patents on the Patent-Scope platform depended on the number of sensors used, where P represents the total number of patents.

Number of Sensors	P = 27
None	25.9%
One sensor	22.22%
Two sensors	33.33%
Three sensors	14.81%
Four sensors	3.70%
Plurality of sensors	0.00%

**Table 6:** Bibliographic references of sensors on the Patent-Scope platform, in alphabetical order.

Sensor	References
Acceleration	[208]
Bio-sensor	[209,210,211,212,213]
Distance/detection	[214]
Encoder	[215,216,217,218,219,220,221,222,223]
Force	[224]
IMU	[210,211,213,214,225]
Pressure	[209,210,211,213,214,215,216,219,223,225,226,227]
Tension	[213,226]
Tilt	[216,227]
Torque	[208,212,224]

**Table 7:** The distribution of the use of pressure sensors in the patents in the Patent-Scope platform, where P represents the total number of patents, and N is the number of patents that use the respective sensor.

Type of Sensor	P = 27
Pressure sensors (N)	12
Foot plantar pressure (N)	12
Gasbag (N)	0
Pneumatic (N)	0

**Table 8:** The distribution of the use of encoders in the patents on the Patent-Scope platform, where P represents the total number of patents, and N is the number of patents that use the respective sensor.

Type of Sensor	P = 27
Encoder, (N)	9
Encoder (angle) (N)	6
Encoder (unspecified type) (N)	2
Encoder (linear, rotary) (N)	0
Encoder (magnetic, photoelectric) (N)	0
Encoder (incremental, absolute (N)	1

**Table 9:** Bibliographic references of sensors on the Lens platform, in alphabetical order.

Sensor	References
Bio-sensor	[228,229,230,231,232,233,234,235,236,237,238,239,240,241,242,243]
Distance/detection	[230,244,245,246,247,248,249]
Encoder	[228,229,230,233,234,235,236,241,243,244,246,247,249,250,251,252,253,254,255,256,257,258,259,260,261,262,263]
Force	[234,238,242,248,250,251,252,254,258,259,260,264,265,266]
Force–torque (F/T)	[231,239,243]
Hall effect	[263]
IMU	[232,233,234,235,236,237,238,239,240,241,243,247,248,251,252,253,254,255,259,261,262,263,267,268,269,270]
Position	[230,242,257,260,262,267,270,271,272]
Potentiometer	[238,241,243,245,251,252,259]
Pressure	[228,229,230,231,232,233,234,236,239,240,241,242,243,244,246,247,249,253,255,256,262,264,267,268,270,271,273,274,275,276]
Speed	[228,242,250,267]
Tension	[231,235,236,239,255,273,276]
Torque	[230,247,256,268,269,276]
Other	[234,238,247,248,255]

**Table 10:** The distribution of pressure sensors in the patents on the Lens platform, where P represents the total number of patents, and N is the number of patents that use the respective sensor.

Type of Sensor	P = 67
Pressure sensors, N (P%)	30 (35.82)
Foot plantar pressure, N (P%)	24 (31.34)
Gasbag, N (P%)	0 (0.00)
Pneumatic, N (P%)	2 (2.97)
Mechanic, N (P%)	1 (1.49)
Fluid, air N (P%)	3 (4.48)

**Table 11:** The distribution of the use of encoders in the patents on the Lens platform, where P represents the total number of patents, and N is the number of patents that use the respective sensor.

Type of Sensor	P = 67
Encoder, N (P%)	27 (40.3)
Encoder (angle), N (P%)	19 (28.36)
Encoder (unspecified type), N (P%)	2 (2.99)
Encoder (linear, rotary), N (P%)	5 (7.46)
Encoder (optical), N (P%)	1 (1.49)

**Table 12:** The distribution of the use of force sensors in the patents in the Lens platform, where P represents the total number of patents, and N is the number of patents that use the respective sensor.

Type of Sensor	P = 67
Force sensors, N (P%)	14 (20.9)
Force (unspecified type), N (P%)	8 (11.95)
Force (FSR), N (P%)	1 (1.49)
Force (load cell), N (P%)	4 (5.97)
Force (pulling), N (P%)	1 (1.49)

**Table 13:** Bibliographic references by alphabetical types of actuating system on the Google Patents platform.

Actuating	References
Artificial muscle (air muscle)	[83,134,167,183,277]
Electrical	[30,31,33,34,36,38,39,41,42,43,44,45,46,48,49,51,52,54,55,56,57,59,60,61,62,64,65,67,69,71,72,73,74,75,76,77,78,80,82,83,84,86,87,89,90,91,94,95,97,98,100,101,102,103,104,105,106,107,108,109,110,111,112,113,115,116,117,119,120,121,123,125,126,127,128,129,131,132,133,134,136,137,138,141,142,143,144,146,147,148,149,150,151,152,153,154,156,157,158,160,161,162,163,166,168,169,170,171,172,173,176,177,181,185,189,190,193,196,200,203,204,205,207,278,279,280,281,282,283,284,285,286,287,288,289,290,291,292,293,294,295,296,297,298,299,300,301,302,303,304,305,306,307,308,309,310,311,312,313,314,315,316,317,318,319,320,321,322,323,324,325,326,327,328,329,330,331,332,333,334,335,336,337,338,339,340]
Electro-hydraulic	[186,198]
Electro-mechanical	[36,37,68,78,81,87,88,92,93,99,118,140,158,180,182,325,341,342,343]
Electro-pneumatic	[103,344]
Hydraulic	[34,35,40,41,47,56,58,62,70,72,75,78,79,80,90,95,96,103,113,115,130,136,139,144,145,146,152,161,163,167,169,178,191,201,283,300,305,313,345,346,347]
Mechanic	[32,33,44,53,55,122,135,159,164,165,170,172,174,179,184,192,202,206,338,348,349,350,351,352,353,354,355,356,357,357,358,359,360,361,362,363,364,365,366,367,368,369,370,371]
Pneumatic	[34,35,40,41,50,51,56,58,62,66,67,70,72,75,78,79,80,85,90,95,106,113,115,136,139,144,158,161,163,169,178,188,194,199,283,300,305,313,319,372,373]
Serial elastic (SEA)	[37,105,114,288,291,297,311]
Other	[45,63,114,170,172,374,375,376]

**Table 14:** Bibliographic references of actuating systems on the Patent-Scope platform, in alphabetical order.

Actuating	References
Electrical	[208,209,210,211,212,213,214,215,216,217,218,219,220,221,223,224,225,226,227,377,378,379,380,381,382,383]
Hydraulic	[222]
Pneumatic	[226]

**Table 15:** Bibliographic references of actuating systems on the Lens platform, in alphabetical order.

Actuating	References
Artificial muscle (air muscle)	[255,276]
Electrical	[228,229,230,231,233,234,235,236,237,238,239,240,241,242,243,244,246,247,248,249,250,251,253,254,255,256,259,260,261,262,263,264,265,266,268,269,270,272,273,274,275,276,384,385,386,387,388,389,390,391,392,393,394,395]
Electro-mechanical	[245,248,249,252,390,396,397,398]
Hydraulic	[237,241,243,261,264,265,266,267,270,394]
Mechanic	[257,391,392,399,400]
Pneumatic	[237,241,243,258,261,264,265,266,267,270,271,394]
Serial elastic (SEA)	[401]
Other	[232,236]

## Data Availability

Not applicable.
